# Tools for assessing the content of guidelines are needed to enable their effective use – a systematic comparison

**DOI:** 10.1186/1756-0500-7-853

**Published:** 2014-11-26

**Authors:** Michaela Eikermann, Nicole Holzmann, Ulrich Siering, Alric Rüther

**Affiliations:** Department Evidence-based Health Services Research, Faculty of Health, Department of Medicine, Institute for Research in Operative Medicine (IFOM), Witten/Herdecke University, Ostmerheimer Str. 200, Building 38, 51109 Cologne, Germany; Department Quality of Health Care, Institute for Quality and Efficiency in Health Care (IQWiG), Im Mediapark 8, 50670 Cologne, Germany

**Keywords:** Practice guidelines as topic/standards, Decision making, Quality in health care, Guideline quality, Guideline appraisal

## Abstract

**Background:**

To ensure that clinical practice guidelines (CPGs) form a sound basis for decision-making in health care, it is necessary to be able to reliably assess and ensure their quality. This results in the need to assess the content of guidelines systematically, particularly with regard to the validity of their recommendations.

The aim of the present analysis was to determine the suitability and applicability of frequently used assessment tools for evidence syntheses with regard to the assessment of guideline content.

**Methods:**

We conducted a systematic comparison and analysis of established tools for the assessment of evidence syntheses (guidelines, systematic reviews, health technology assessments). The tools analyzed were: ADAPTE, AGREE II, AMSTAR, GLIA and the INAHTA checklist. We analyzed methodological steps related to the assessment of the reliability and validity of guideline recommendations. Data were extracted and analyzed by two persons independently of one another.

**Results:**

Widely used tools for the methodological assessment of evidence syntheses are not suitable for a comprehensive content-related assessment. They remain mostly at the level of assessment of the documentation of processes. Some tools assess selected content-related aspects, but operationalization is either unspecific or lacking.

**Conclusion:**

None of the tools analyzed enables the structured and comprehensive assessment of the content of guideline recommendations with special regard to their reliability and validity. All tools contribute towards the judicious use of evidence syntheses by supporting their systematic development or assessment. However, further progress is needed, particularly with regard to the assessment of content quality. This includes comprehensive operationalization and documentation of the assessment process to ensure reliability and validity, and therefore to enable the effective use of trustworthy guidelines in the health care system.

**Electronic supplementary material:**

The online version of this article (doi:10.1186/1756-0500-7-853) contains supplementary material, which is available to authorized users.

## Background

According to the current definition by the Institute of Medicine (IOM), clinical practice guidelines (CPGs) “are statements that include recommendations intended to optimize patient care that are informed by a systematic review of evidence and an assessment of the benefits and harms of alternative care options” [1]. They are viewed as tools for making health care decisions more rational, with the ultimate aim of improving the quality and effectiveness of care [2].

To ensure that guidelines form a sound basis for decision-making and standards in health care, it is necessary to be able to reliably assess and ensure their quality. Although methods for guideline development are being further elaborated [1, 3, 4], there still seems to be a need to increase adherence to these standards [5]. Guidelines still show substantial differences in their development process, reporting, methodological quality and, not least, in content [6–13], and many recommendations are based on low-quality evidence [14, 15]. Furthermore, the crucial issue of managing conflicts of interest of guideline panel members has so far not been sufficiently resolved [5, 15]. In addition to the assessment of the development methods, the current inadequacies result in the need to assess the content of guidelines systematically with regard to the appropriate implementation of methodological standards and particularly to the reliability of their recommendations.

In the last few years there have been numerous initiatives to improve the quality of guidelines. As a result, various tools with different objectives have been created in the fields of both guideline development and assessment. In respect of guideline development and adaptation, the activities of the Grading of Recommendations Assessment, Development and Evaluation (GRADE) Working Group and the ADAPTE Collaboration are of particular note [4, 16–19]. A range of tools are available to assess the quality of guidelines. We identified 40 different tools in a systematic search [20]. The one most widely used internationally is the AGREE instrument (Appraisal of Guidelines for Research & Evaluation) [21] and its revised version, AGREE II [22–24]. In addition, a translated and amended version of AGREE, the German Instrument for Methodological Guideline Appraisal (DELBI) [25], is available for use in the German health care system. However, the tools focus on methodological issues around guideline development and reporting, and none of them appears to be suited to conduct a complete, systematic and content-related analysis of guideline recommendations, which seems to be essential to ensure that recommendations are reliable and valid [26, 27].

There is still a need for development here, especially as high methodological quality does not necessarily correlate with high content quality [28]. To provide tools for the assessment of the content of guidelines, particularly concerning the validity of their recommendations, the development of a further assessment tool therefore suggests itself. However, in view of the effort involved, it would be meaningful first to examine systematically to what extent an assessment of content quality can be conducted by means of existing tools for the assessment of evidence syntheses, in particular guidelines, but also systematic reviews or Health Technology Assessments (HTAs).

The aim of the present analysis was to determine the suitability and applicability of frequently used assessment tools for evidence syntheses with regard to the assessment of guideline content, namely, the appropriate implementation of methodological standards and particularly the reliability of recommendations.

## Methods

The present paper is based on the following definitions by the IOM:

*Validity:* Practice guidelines are valid if, when followed, they lead to the health and cost outcomes projected for them, with other things being equal. A prospective assessment of validity will consider the projected health outcomes and costs of alternative courses of action, the relationship between the evidence and recommendations, the substance and quality of the scientific and clinical evidence cited, and the means used to evaluate the evidence [1].

***Reliability/Reproducibility:*** Practice guidelines are reliable and reproducible: (1) if—given the same evidence and methods for guidelines development—another set of experts would produce essentially the same statements; and (2) if—given the same circumstances—the guidelines are interpreted and applied consistently by practitioners or other appropriate parties. A prospective assessment of reliability may consider the results of independent external reviews and pretests of guidelines [1].

### Assessment tools analyzed

We conducted a systematic comparison and analysis of selected established tools for the development and assessment of evidence syntheses. On the basis of a systematic search from another project [20] we included the following guideline-specific tools: ADAPTE (assessment module from the ADAPTE Manual and Toolkit) [16], AGREE II (Appraisal of Guidelines for Research and Evaluation) [22, 23] and GLIA (GuideLine Implementability Appraisal) [29, 30]. Furthermore, we included AMSTAR (A Measurement Tool to Assess Systematic Reviews) [31] and the INAHTA checklist [32, 33] as assessment tools for systematic reviews and HTAs. This is because our main focus was on the appropriate implementation of methodological standards, which can also be an issue in systematic reviews or HTAs. Besides this the inclusion of these tools in our analysis was suggested by guidelines experts in numerous discussions on conferences or internal workshops.

Due to the numerous tools available for the assessment of evidence syntheses [20, 34, 35], we decided to focus the analysis on the current, established and most commonly used ones, which we identified in the context of our previous review [20] and which are mostly validated (Additional file 1). They are often based on or represent further developments of former tools; an analysis of former tools therefore seemed superfluous. Furthermore, a complete analysis of all available tools is not feasible within an acceptable period of time and with an acceptable use of resources.

### Analysis criteria

We summarized aspects regarding the assessment of content quality, which are already integral parts of the commonly used assessment tools, and which could form the basis for the development of tools for the assessment of guideline content. We analyzed all methodological steps relating to the assurance or assessment of the validity of guidelines or guideline recommendations. We made no detailed analysis of methodological steps essentially related to external factors influencing guideline validity; for example, we did not check the suitability of a recommendation in a certain context or the correctness of the Grade of Recommendation (GoR) awarded.

The following categories of the tools were analyzed:

- Characteristics of the tools
  - description
  - rationale for development
  - objective(s)
  - answer and evaluation categories
  - documentation of the assessment
  - consequences of the assessment

- Components and operationalization of the assessment. Assessment of the:
  - medical definitions
  - thematic completeness
  - unambiguity of the content of the recommendation
  - outcomes applied (especially with regard to completeness and patient relevance)
  - literature search and study selection
  - evaluation and interpretation of the evidence base
  - consensus process

Data were extracted and analyzed by two persons independently of one another. Disagreements were resolved by discussion. For each item we analyzed whether the tool assessed the documentation of guideline development as well as the content (by means of the appropriate implementation of methods and the appropriateness of the results). We also checked whether the steps for the assessment of content were fully operationalized. We defined “operationalization” as any information or guidance given within the tools on how to assess the relevant item (e.g. examples, instructions, rating matrices).

## Results

### Characteristics of the tools analyzed

The tools serve different purposes but have a common goal, i.e. to ensure the high quality of guidelines or other evidence syntheses (see Table 1).

**Table 1:** **Characteristics of the tools analyzed**

	ADAPTE	AGREE II	GLIA	AMSTAR	INAHTA
**Basic characteristics**					
Specific tools for assessment of evidence synthesis	**X**	**X**	**X**	**X**	**X**
Guideline-specific tools (development or assessment)	**X**	**X**	**X**	**−**	**−**
Primary objective: assessment of guidelines	**in part**	**X**	X*	**−**	**−**
Validated tool	**X**	**X**	**X**	**X**	**−**
Individual recommendation or question is focus of the assessment	**X**	**−**	**in part**	**X**	**X**
Extraction of data to be evaluated	**in part**	**−**	**−**	**−**	**−**
Uniform format for answers	**in part**	**X**	**in part**	**X**	**in part**
Explicit definition of “quality”	**X**	**X**	**−**	**X**	**−**
Explicit definition of “validity”	**X**	**−**	**X**	**−**	**−**
**Assessment of quality**					
Aspects of methodological quality	**X**	**X**	**X**	**X**	**X**
Content-related aspects	**X**	**In part** ^**†**^	**X**	**−**	**X**
Quality of the evidence base	**X**	**In part** ^**†**^	**In part**	**−**	**−**
**Consequences of the assessment**					
Consequences for use of evidence base	**X**	**−**	**−**	**−**	**−**
Consequences for use of the whole guideline (e.g. recommendation for use, suitability as a source)	**X**	**X**	**X**	**n. a.**	**n. a.**
Consequences for use of guideline recommendation or conclusion of a systematic review / HTA (e.g. modification when used as a source)	**X**	**−**	**X**	**−**	**−**

*: with special focus on implementability.

†: content-related aspects (e.g. regarding the evidence base of the recommendations) are given as instructions to fill in the assessment form.

AMSTAR and the INAHTA checklist are not targeted towards guidelines, but are tools for assessing systematic reviews or HTA reports. AGREE II and AMSTAR are tools for a structured assessment of guideline quality. ADAPTE has a special status, as it is a tool for guideline adaptation, i.e. for the development of new guidelines on the basis of pre-existing guidelines produced in a different setting, and contains methods for their assessment. GLIA is a tool for the assessment of the implementability of guidelines. AGREE II, ADAPTE, GLIA and AMSTAR have been validated [22, 23, 30, 31, 36–38].

The tools differ in their level of analysis. A distinction can be made here as to whether they relate to the assessment of a whole guideline/systematic review or to an individual recommendation or question. Assessments using AGREE are consistently made at the level of the whole guideline, while ADAPTE and GLIA are applied completely or largely to an individual recommendation or question.

The extraction of the data to be evaluated is not conducted in a uniform manner. No tool specifies the full extraction of data; ADAPTE specifies partial extraction. This does not apply to AGREE II, AMSTAR, GLIA and the INAHTA checklist, where only the assessment itself is documented and, if required, supplemented by comments.

Not all tools analyzed show a uniform format for answers. For example, the assessment with AGREE II is conducted by means of Likert scales, AMSTAR offers 4 possible answers, and ADAPTE specifies several possible answers for the individual assessment steps (e.g. yes/no/unclear; 4-stage Likert scale: strongly agree – strongly disagree.

### Definition of quality

An explicit definition of quality is given in 2 tools (AGREE and AMSTAR). They congruently name the prevention of systematic errors in the development of guidelines or systematic reviews as a quality criterion.

### Definition of validity

Only ADAPTE and GLIA provide definitions for the various validity terms. Whereas ADAPTE defines scientific validity (consistency between evidence, its interpretation and recommendations), GLIA defines validity as the degree to which the recommendation reflects the intent of the developer and the strength of the evidence.

### Assessment areas

According to the different objectives of the tools, various aspects of the quality of a guideline/recommendation or systematic review are captured.

In ADAPTE, the methodological assessment of guidelines is conducted, among other things, with AGREE. Accordingly, AMSTAR can be used to assess the methodological quality of a systematic review. An assessment of content-related aspects is also conducted in the ADAPTE manual as well as to a very limited extent in AGREE II and GLIA. The quality of evidence as such is examined neither in the assessment of guideline quality with AGREE II, nor in the assessment of the quality of systematic reviews with AMSTAR. All guideline-specific tools contain questions on the acceptance and applicability of the guideline or recommendation.

### Consequences of the assessment

In 3 of the 5 tools analyzed, the consequences that may result from the assessment are described. AMSTAR and the INAHTA checklist provide no information in this respect. As shown in Table 1, an assessment with AGREE II may lead to the rejection of guidelines or recommendations or to their adoption with limitations. The assessment with GLIA results in consequences related to the focus of the tool: the implementability of a guideline.

### Components and operationalization of the assessment

In a second step we analyzed the components of the assessment, as well as the operationalization of the assessment process. The analysis was performed from the perspective of guideline assessment (see Table 2).

**Table 2:** **Content and operationalization of the assessment**

		ADAPTE	AGREE II	GLIA	AMSTAR	INAHTA-checklist
Assessment of the medical definitions used in the guideline or systematic review of interest	Assessment of documentation	x	x	x	−	−
	Assessment of content	−	−	−	−	−
	Complete operationalization^*^	n. a.	n. a.	n. a.	n. a.	n. a.
Assessment of the unambiguity of content of the guideline recommendation or conclusion of the systematic review/HTA	Assessment of content	(x)^‡,§^	(x)^‡^	x	−	−
	Complete operationalization	x^‡^	x	−	n. a.	n. a.
Assessment of outcomes considered	Assessment of documentation	(x)^§,||^	(x)^||^	−	x	−
	Assessment of content – completeness	−	−	−	−	−
	Assessment of content – patient relevance	x	−	−	−	−
	Complete operationalization^*^	−	n. a.	n. a.	n. a.	n. a.
Assessment of the literature search	Assessment of documentation	x	x	−	x	x
	Assessment of content – currency	x	−	−	−	−
	Assessment of content – completeness^**†**^	x	x	−	−	−
	Assessment of content – plausibility	x	x	−	−	−
	Complete operationalization^*^	−	-	n. a.	n. a.	n. a.
Assessment of study selection	Assessment of documentation	x	x	−	x	x
	Assessment of content	x	x	−	x	−
	Complete operationalization^*^	−	−	n. a.	−	n. a.

*: Regarding the assessment of content-related aspects. An item is rated as n.a. if no assessment of content is provided by the tools.

†: “Complete” also refers to the consideration of unpublished data.

‡: An assessment of the unambiguity of content of the totality of recommendations is made.

§: The assessment is made within the context of the AGREE assessment as a component of the ADAPTE toolkit.

||: Only indirect assessment of the documentation of outcomes.

*Abbreviations:* n. a.: not applicable.

For every criterion we checked whether an assessment of the documentation, as well as of content, was planned. If the latter case applied, we analyzed whether the complete operationalization of the process was specified in the tool analyzed.

### Assessment of medical definitions and unambiguity of content

The basis of the assessment of the unambiguity and quality of the content of guideline recommendations is the clear classification of patients, interventions and outcomes according to the PICO formula. All definitions relevant in this context must be clearly explained.

In AGREE II, ADAPTE and GLIA the assessment of the medical definitions used in the guideline/recommendation of interest is limited to an evaluation of their documentation. AMSTAR assesses whether the characteristics of the studies included in the systematic review are presented; it does not evaluate the documentation and unambiguity of content of the definitions used in the systematic review itself.

AGREE II only assesses the unambiguity of content for the totality of recommendations in a guideline. ADAPTE does not include an assessment going beyond this, nor does AMSTAR assess the unambiguity of content of the conclusions drawn in the systematic review of interest.

### Assessment of the outcomes considered

The medical benefit of an intervention should relate to the patient and therefore should ideally be assessed on the basis of patient-relevant outcomes [39]; such outcomes should therefore preferably be assessed and reported in guideline recommendations.

None of the assessment tools included fully assess the completeness and patient relevance of outcomes. AGREE II indirectly assesses the documentation of outcomes considered in the guideline (aim of the guideline, key questions, monitoring and/or auditing criteria). However, no complete operationalization is given for the definition of relevant outcomes. ADAPTE also assesses the clinical relevance of outcomes but does not specify how the process is operationalized.

### Assessment of the literature search and study selection

A systematic literature search is a key factor in the preparation of high-quality evidence syntheses such as CPGs or systematic reviews. Errors in the search strategy may lead to incomplete identification of the relevant literature [40]. The same applies to the erroneous exclusion of publications during the study selection process.

With respect to the literature search, we analyzed whether the tools included an assessment of the components of the search strategy applied, especially concerning currency, completeness and plausibility. Such a comprehensive assessment is prescribed in ADAPTE, but it is not specified how this process is operationalized. AMSTAR only describes the documentation of the search, but does not assess the components of the strategy itself. AGREE II assesses the appropriateness of search strategies but does not provide any further operationalization.

In respect of the completeness of the search, special attention should be paid to whether unpublished data were searched for. Limitation to published data may lead to considerable bias in the evaluation of the evidence [41].

No tool explicitly described how to handle unpublished data. However, AMSTAR assesses how potential publication bias is considered in guidelines and systematic reviews.

All tools, with the exception of GLIA, include questions on the documentation of study selection. AMSTAR also addresses the systematic exclusion of literature by means of publication type. Additionally, ADAPTE assesses the suitability of inclusion and exclusion criteria, but does not specify how this process is operationalized.

### Assessment of the quality rating of the evidence base

The guideline or review authors’ quality rating of the evidence base covers the evaluation and interpretation of the literature underlying the respective recommendation or conclusion.

ADAPTE, GLIA and AMSTAR include questions on how the quality of the evidence base is rated and on the internal consistency between evidence base and recommendations. These relate in part to an assessment of documentation, but also in varying depth of detail to aspects of content.

However, for many points the assessment tools fail to mention how the process is operationalized. AGREE II makes only a general assessment for the whole guideline or, more specifically, for the whole body of evidence as to whether benefits and risks were considered in the formulation of the recommendations or the strength and limitations of the body of evidence.

### Assessment of the consensus process

The consensus process is an elementary component in the generation and formulation of guideline recommendations. Especially in cases where evidence is lacking or conflicting evidence is available, and recommendations are made or grades of recommendation allocated on the basis of a consensus decision, a properly conducted consensus process is essential.

ADAPTE and AGREE II include questions on the documentation of the consensus process. This criterion is not applicable to AMSTAR and the INAHTA checklist.

AGREE II, ADAPTE, AMSTAR, GLIA and the INAHTA checklist are tools that can contribute towards improving the quality of guidelines and other evidence syntheses, such as systematic reviews or HTA reports, by supporting the systematic development or assessment of these publications. The tools analyzed are not suitable for a comprehensive assessment of the content of guidelines or other evidence syntheses, and often remain at the level of the assessment of the documentation of processes. Further evaluation in the sense of an assessment of content with regard to the reliability and validity of recommendations and conclusions is only performed to a limited degree or is lacking. In addition, the operationalization of the assessment process is either unspecific or completely absent. Nevertheless, with the development of AGREE II, including the addition of item 9. “The strength and limitations of the body of evidence are clearly described”, an important step was taken towards a more content-related assessment and therefore towards an assessment of guideline reliability and validity.

## Discussion

We conducted a systematic comparison and analysis of established tools for the assessment of evidence syntheses to identify components for content assessment. Our results were to support the development of comprehensive tools for content assessment. Basically, the question can be posed as to why an assessment of guideline content and other secondary literature is necessary at all, and whether an assessment of methodological quality would be sufficient. One reason is that, even though requirements and recommendations for guideline development, as well as tools for the assessment of methodological quality, have existed for some years, guideline recommendations and systematic reviews on comparable questions vary widely [13, 42]. Especially when system decisions are based on guideline recommendations, it should be ensured that these recommendations form a sound basis for decision-making, which at least necessitates the assessment of their content.

Some guideline assessment tools, such as AGREE II, require an independent external review in order to improve guideline quality. The IOM describes the external review as one of the standards for trustworthy CPGs [1]. Furthermore, a description of the methodology used to conduct the external review should be presented, for example, in AGREE II [24]. This seems to be a crucial point: How to perform an external review of guideline content only on the basis of the documentation of guideline methodology and without standards for content assessment.

The analysis presented here forms part of the further development of guideline assessment tools with a focus on the assessment of guideline content. The analysis criteria examined were defined within the framework of this development. We decided to focus on the identification, selection and interpretation of the evidence. Nevertheless, other aspects may influence the reliability and validity of guidelines and the interpretation of evidence, for example, the handling of competing interests of guideline panel members. Established tools were specifically chosen for this analysis. A comparison such as the one performed can serve to highlight both differences and new approaches. However, it is not suited to examine whether all relevant criteria for the assessment of guideline content were actually considered, especially since the analysis criteria selected have so far not been discussed with external researchers. The tools presented in this paper differ in their objectives. Therefore the absence of certain components cannot be generally viewed as a deficit of these tools, as this is not only due to the different objectives but partially to variations in requirements, especially concerning applicability. In particular, this should be viewed against the background that none of the tools analyzed was developed to explicitly address the assessment of guideline content. Nevertheless, it is surprising that the assessment of guideline content still plays a subordinate role compared to the assessment of guideline methodology, even though the limitations of a purely methodological assessment have been known for years [11, 27].

Individual aspects of guideline quality, such as the identification and inclusion of unpublished data, have become increasingly important in recent years, but have so far been insufficiently addressed in the tools analyzed. It is to be expected that this issue will be addressed in assessment tools in the future.

Reliability and validity may vary between the different key questions and recommendations. It is therefore surprising that up to now, most guideline assessment tools focus on the whole guideline, instead of on the single recommendations or key questions.

The main limitation of our analysis is that we did not analyze all available assessment tools for guidelines and other secondary literature. On the basis of our systematic search for guideline assessment tools [20] and on an HTA report on quality assessment tools [34, 35], we identified 40 tools for guideline assessment and 15 for the assessment of systematic reviews. A comprehensive data extraction and analysis of all these tools would have been far beyond our resources. Nevertheless, we analyzed the established tools most commonly used in their specific area.

## Conclusion

None of the tools analyzed enables the structured and comprehensive assessment of the content of guideline recommendations with special regard to their reliability and validity. Those available are almost exclusively designed to assess guidelines at the level of the development process and to assess the documentation of this process. There is thus a need for further progress here. The approach to be adopted should be compatible with existing tools in the field of guideline development and assessment and should close gaps, particularly with regard to the comprehensive operationalization and documentation of the assessment process. Driven by idealistic concepts, developers and users of CPGs need practically applicable tools for the assessment of guideline content to ensure reliability and validity and therefore to enable the effective use of guidelines in the health care system.

## Electronic supplementary material

Additional file 1:**Extraction Tables.**(DOC 166 KB)
