# Supplementary material for: Tools for assessing the content of guidelines are needed to enable their effective use – a systematic comparison
Source: BMC Res Notes. 2014 Nov 26;7:853. doi: 10.1186/1756-0500-7-853 (PMC4258382; doi:10.1186/1756-0500-7-853)
Supplement: Supplementary file 1 — Additional file 1:Extraction Tables.(DOC 166 KB) [file 13104_2014_3375_MOESM1_ESM.doc]

Additional file 1: Extraction Tables

Table 1: Data Extraction: Characteristics of analyzed tools

| **ADAPTE** | **AGREE II** | **GLIA (version 2.0)** | **AMSTAR** | **INAHTA Checklist** |
| --- | --- | --- | --- | --- |
| **Description of tools** | | | | |
| Systematic approach for the adaptation of guidelines produced in one setting to be used in a different cultural organizational context.  Manual and Resource Toolkit | Validated instrument to assess the methodological quality of clinical practice guidelines as well as the reporting of the guideline development process | Tool to assess the implementability of clinical practice guidelines. | Validated instrument to assess the methodological quality of systematic reviews (of randomized controlled trials)  Note: A new tool based on the current AMSTAR, but validated against observational studies is currently under development | Checklist to assess the methodological quality of an HTA report. |
| **Object of analysis:**  Complete guideline and single recommendations | **Object of analysis:**  Complete guideline; no analysis of single recommendation | **Object of analysis:**  Complete guideline (questions 1-9) and single recommendations (Questions 10-30) | **Object of analysis:**  Systematic review | **Object of analysis:**  HTA report |
| **Rationale for development:**   - Development and updating of high-quality clinical practice guidelines require substantial resources. - Pressure for organizations to produce guidelines rapidly, to ensure medical practice is consistent with current, emerging medical knowledge and with increasingly limited resources. - Guideline adaptation (modification of a guideline produced in one cultural and organizational setting for application in a different context) is proposed to take advantage of existing guidelines and reduce duplication of effort.   🡪 might be used as an alternative to de novo guideline development | **Rationale for development:**   - The potential benefits of guidelines are only as good as the quality of the guidelines themselves. - Appropriate methodologies and rigorous strategies in the guideline development process are important for the successful implementation of the resulting recommendations - The quality of guidelines can be extremely variable - The AGREE Instrument was developed to address the issue of variability in guideline quality.   🡪 A tool that assesses the methodological rigor and transparency in which a guideline is developed. | **Rationale for development:**   - Implementation refers to that part of the guideline lifecycle in which systems are introduced to influence clinicians' behaviour toward guideline adherence. - Some guidelines have been found to be more difficult to put into practice than others. | **Rationale for development:**   - High methodological quality is a pre-requisite for valid interpretation and application of review findings. - Systematic reviews may differ in quality, and yield different answers to the same question. - No agreement on which instrument to use when measuring the quality of systematic reviews | **Rationale for development:**   - HTA reports vary considerably in their depth and scope of analysis, given differences in the types of problem being addressed, policy requirements and the time and resources available for assessment. - Readers of an HTA report need to be able to easily obtain information on the purpose of the assessment, the methods used, assumptions made and conclusions reached. |
| **Objectives:**  Development of a systematic approach to aid in the adaptation of guidelines based on:   - Evidence-based principles for guideline development - Reliable and consistent methods to ensure the quality of the adapted guideline - Participation of key stakeholders to foster acceptance and ownership of the adapted guideline - Explicit consideration of context to ensure relevance for local practice and policies - Transparent reporting to promote confidence in recommendations - Flexible format to accommodate specific needs and circumstances - Respect for and acknowledgement of source guidelines material. | **Objectives:**  The purpose of the AGREE II, is to provide a framework to:   - assess the quality of guidelines; - provide a methodological strategy for the development of guidelines; - inform what information and how information ought to be reported in guidelines. | **Objectives:**  To provide information about a guideline’s implementability by identification of intrinsic barriers in order to provide information:   - to guideline authoring groups, which may decide to modify content to improve implementability - to guideline implementers to anticipate potential problems in implementation. | **Objectives:**  To develop a new instrument for assessing the methodological quality of systematic reviews by building upon empirical data collected with previously developed tools and utilizing expert opinion. | **Objectives:**  To improve the quality of HTA reports.   - Aid to furthering a consistent and transparent approach to health technology assessment. - Clear identification of what has been done in an HTA report and of any significant limitations. - Improving the usefulness and generalizability of HTA reports by promoting transparency in the assessment process. |
| **Definition of ”quality of recommendations” / ”guideline quality”** | | | | |
| Glossary: Definition of “guideline quality” referring to AGREE | - Quality of guidelines:   The confidence that the potential biases of guideline development have been addressed adequately and that the recommendations are both internally and externally valid, and are feasible for practice  (mentioned in the paragraph “history of the AGREE-project but no refined definition is given for AGREE II) | No explicit definition. | n.a. Quality of systematic reviews: The likelihood that the design of a systematic review will generate unbiased results. | n.a.  No explicit definition. Of HTA quality. |
| **Definition of “validity of recommendations” / “guideline validity”** | | | | |
| No explicit definition  Within Tool 14 „scientific validity of guidelines“ is described as consistency between evidence, its interpretation and recommendations | No explicit definition | The degree to which the recommendation reflects the intent of the developer and the strength of evidence | n.a.  No explicit definition of SR validity. | n.a.  No explicit definition of HTA validity. |
| **Answer and evaluation categories** | | | | |
| - Assessment of (methodological) guideline quality. For the assessment of guideline quality the AGREE-Instrument is used (Tool 9). - Assessment of guideline currency (Step 12) - Assessment of guideline content (Step 13) - Assessment of guideline consistency (Step 14) - Search and selection of studies (Tool 13) - Links between evidence and recommendations (Tool 14) - Assessment of applicability of guideline/recommendations (Step 15)) | 23 key items organized within 6 domains   - Scope and Purpose - Stakeholder Involvement - Rigour of Development - Clarity of Presentation - Applicability - Editorial Independence   The 6 domains are followed by 2 global rating items (“Overall Assessment”). | 30 items arranged into 9 dimensions:   - Global considerations - Executability (exactly what to do) - Decidability (precisely under what conditions to do something) - Validity (degree to which the recommendation reflects the intent of the developer and the strength of evidence) - Flexibility (degree to which a recommendation permits interpretation and allows for alternatives in its execution) - Effect on process of care (degree to which the recommendation impacts upon the usual workflow in a typical care setting) - Measurability (degree to which the guideline identifies markers or endpoints to track the effects of implementation of the recommendation) - Novelty/innovation (degree to which the recommendation proposes behaviors considered unconventional by clinicians or patients) - Computability (ease with which a recommendation can be operationalized in an electronic information system) only applicable when an electronic implementation is planned | 11 single items The iitems were not organized in domains. | The checklist includes 14 items organized within 5 domains:   - Preliminary - Why? - How? - Context? - What then? |
| **Documentation of the assessment** | | | | |
| Documentation into evaluation sheets (Toolkit: Assessment tables and examples)   - Different answer categories for the assessment steps, e.g. yes/no(/unsure); likert-scales - Only partial extraction of data - Only partial operationalization of assessment steps | - Each of the AGREE II items and the two global rating items are rated on a 7-point scale (1–strongly disagree to 7–strongly agree). - Operationalization of assessment steps: The User’s Manual provides guidance on how to rate each item using the rating scale and also includes 3 additional sections to further facilitate the user’s assessment. The sections include User’s Manual Description, Where to Look, and How to Rate. - No mandatory extraction of assessed data but field for comments. | - Assessments tables - Appraisers should answer each question with one of the following responses: Y The recommendation meets this criterion fully, N The recommendation does not meet this criterion,? Rater is unable to address this question because of insufficient knowledge or experience in this area, N/A Criterion is not applicable to this recommendation” - Question 1-9 (global considerations): guideline specific assessment and documentation. - Question 10-30: recommendation-specific assessment and documentation. - No mandatory extraction of assessed data but field for comments. | Assessment and documentation into assessment sheets   - 4 options for answering (yes, no, can't answer or not applicable) - No extraction of assessed data. - Criteria given to standardize assessment but not fully operationalized - No mandatory extraction of assessed data | Documentation into a summary form / checklist.   - Different answer categories for the assessment steps, e.g. yes/no/partly; tick a box - No mandatory extraction of assessed data |
| **Consequences of the assessment** | | | | |
| **5 options for the use of assessed guidelines:**   - Rejection of the whole guideline - Acceptance of the whole guideline and all of its recommendations - Acceptance of the evidence summary of the guideline and rejection of the interpretation of evidence and the recommendations - Acceptance of specific recommendations and rejection of others. - Modification of specific recommendations (e.g. addition of new data, change of the wording) | - A quality score is calculated for each of the six AGREE II domains. - Calculating domain scores: - Summing up all the scores of the individual items in a domain and by scaling the total as a percentage of the maximum possible score for that domain. - Interpreting Domain Scores: - Domain scores are independent and should not be aggregated into a single quality score. - No predefined minimum domain scores or patterns of scores across domains to differentiate between high quality and poor quality guidelines. (decision should be made by the user and guided by the context) - Overall assessment: - Judgment on to the quality of the guideline, taking into account the criteria considered in the assessment process. - Judgement whether the use of the guideline is recommended. | Summary report on Barriers, specifics, suggested remedy and resolution.   - An examination of the barriers recorded on the summary report should provide an understanding of impediments to implementation of the guideline statement. - A conditional recommendation that fails any decidability or executability criterion will be impossible to implement as stated. - Likewise, an imperative that fails any executability criterion will not be implementable. - Developers may choose to make modifications to the guideline document before disseminating the guideline. - Implementers can target their efforts toward addressing identified barriers. | - No consequences of the assessment described. - Overall quality score, no cut-off-values provided. | - No consequences of the assessment described. - No overall assessment (with or without cut-off-values) provided. |

Table 2: • Components and operationalization of the assessment.

| **ADAPTE** | **AGREE II** | **GLIA** | **AMSTAR** | **INAHTA Checklist** |
| --- | --- | --- | --- | --- |
| **Assessment of the medical definitions** | | | | |
| - No further assessment of relevant medical definitions - Development of key questions and comparison with source guideline based on **PIPOH-**items - Population - Intervention(s) - Professionals/patients (audience for whom the guideline is prepared) - Outcomes - Healthcare setting and context | - No further assessment of relevant medical definitions - Assessment of specific description of the health questions based on setting, population, intervention, comparator(s), and outcomes (PICOS) - Assessment of specific description of the population covered by the guideline:   Are the following criteria defined?   - target population, gender and age - clinical condition (if relevant) - severity/stage of disease (if relevant) - comorbidities (if relevant) - excluded populations (if relevant) | - No further assessment of relevant medical definitions - Assessment of specific description of the health questions   Are the following criteria defined?   - target patient population - setting in which the guideline is used | - No further assessment of relevant medical definitions - Assessment of the characteristics of the included studies regarding: - population - intervention(s) - outcome(s) | - No further assessment of relevant medical definitions |
| **Assessment of the thematic completeness** | | | | |
| Screening of potential source guideline if the topic of interest is addressed. No further assessment of the thematic completeness. | No overall assessment of thematic completeness  No assessment of thematic completeness regarding the guideline topics | No overall assessment of thematic completeness  No assessment of thematic completeness regarding the guideline topics | n.a. | n.a. |
| **Assessment of the unambiguity of the content of the recommendation / the conclusion** | | | | |
| s. AGREE  No further assessment of the unambiguity of the content of the recommendations | - Assessment of the unambiguity of the content of the body of recommendations   (Item 15: The recommendations are specific and unambiguous)  Item content includes the following criteria:   - statement of the recommended action - identification of the intent or purpose of the recommended action (e.g., to improve quality of life, to decrease side effects) - identification of the relevant population (e.g., patients, public) - caveats or qualifying statements, if relevant (e.g., patients or conditions for whom the recommendations would not apply)   Additional considerations:   - In the event of multiple recommendations (e.g., management guidelines), is there clarity regarding to whom each recommendation applies? - If there is uncertainty in the interpretation and discussion of the evidence, is the uncertainty reflected in the recommendations and explicitly stated? | - Global consideration - Item 7: Is the guideline internally consistent, i.e., without contradictions between recommendations or between text recommendations and flowcharts, summaries, patient education materials, etc.? - Dimension “Executability (exactly what to do)”: - Item 10: Is the recommended action (what to do) stated specifically and unambiguously? - Item 11: Is sufficient detail provided or referenced (about how to do it) to allow the intended audience to perform the recommended action   🡪 not fully operationalized   - Dimension “Decidability (precisely under what conditions (e.g., age, gender, clinical findings, laboratory results) to do something)”: - Item 12: Would the guideline's intended audience consistently determine whether each condition in the recommendation has been satisfied? - Item 13: Are all reasonable combinations of conditions addressed? - Item 14: If this recommendation contains more than one condition, is the logical relationship (ANDs and ORs) between conditions clear?   🡪 not fully operationalized | - No assessment of the unambiguity of the conclusions of the systematic review. | - Assessment if the conclusions from the HTA are clearly stated. |
| **Assessment of the outcomes applied** | | | | |
| - Assessment of documentation (s. AGREE) - No further assessment of the completeness of outcomes regarded in the source guideline - Examples but no complete operationalization for the definition or relevant outcomes given - Differentiation in - Patients outcomes - System outcomes - Public health outcomes - Examples but no complete operationalization for the assessment of clinical relevance of outcomes | - No further assessment of the completeness of the outcomes - Within item 9 (Strengths and limitations of the body of evidence are clearly described) one of the assessment criteria is the appropriateness/relevance of primary and secondary outcomes considered   🡪 no further operationalization | - No assessment of the completeness and patient relevance of the outcomes | - No assessment of the completeness and patient relevance of the outcomes - Assessment of the documentation of outcomes assessed in the included studies. | - No assessment of the completeness and patient relevance of the outcomes |
| **Assessment of literature search and study selection** | | | | |
| - Assessment of guideline currency: - Rapid review of literature - Contact the guideline developer for further information - Literature search on relevant websites with up to date informations - Contact experts - Screening for alerts on the intervention of interest - Assessment of identification and selection of evidence - Assessment of the appropriateness of databases and websites searched   🡪 no further operationalization   - Assessment of the comprehensiveness of the search (publication years, languages, keywords, search methods)   🡪 partly operationalized   - Assessment of the documentation of the search (search strategy) - Assessment of the documentation of study selection process - Assessment of appropriateness of inclusion- and exclusion criteria   🡪 no further operationalization   - Assessment of conformity of study selection with predefined inclusion- and exclusion criteria   🡪 no further operationalization   - No assessment of the use of unpublished data | - Item 7. Systematic methods were used to search for evidence:   Item content includes the following criteria:   - named electronic database(s) or evidence source(s) where the search was performed - time periods searched - search terms used - full search strategy included   Additional considerations:   - Is the search relevant and appropriate to answer the health question? (e.g., all relevant databases and, appropriate search terms used   🡪 no further operationalization   - Is there enough information provided for anyone to replicate the search? - Item 8. The criteria for selecting the evidence are clearly described.   Item content includes the following criteria:   - description of the inclusion criteria, including target population (patient, public, etc.) characteristics, study design, comparisons (if relevant), outcomes, language (if relevant), context (if relevant) - description of the exclusion criteria (if relevant)   Additional considerations:   - Is there a rationale given for the chosen inclusion/exclusion criteria? - Do inclusion/exclusion criteria align with the health question(s)?   🡪 no further operationalization   - Are there reasons to believe that relevant literature may not have been considered? - No assessment of the inclusion of unpublished data | - No assessment of literature search and study selection | - Assessment of the documentation of: - databases used - time periods searched - Key words and/or MESH terms respectively provided search strategy - No critical appraisal of the search strategy - Assessment of the status of publication (i.e. grey literature) as an inclusion criterion - Assessment of duplicate study selection and data extraction - Assessment of the documentation of included and excluded studies - Assessment of the assessment of publication bias - No further assessment of the inclusion of unpublished data. | - Assessment of the provided information regarding: - Search strategy - Databases - time periods searched - language restrictions - primary data - other kind of information resources - complete lists of included and excluded studies - Inclusion and Exclusion criteria |
| **Assessment of the evaluation of the evidence base** | | | | |
| Scientific validity of guidelines (Consistency between selected evidence, its interpretation and resulting recommendations   - **Validity of evidence:** - Low risk that relevant evidence is missed - Explicit criteria for selecting the evidence - Setting and protocols of selected studies fit with the health questions - Criteria for assessing the evidence are adequately reported - Low risk of biased evidence included in guideline - Outcomes were clinically sound - Appropriate statistical analysis (especially meta-analysis)   🡪 no further operationalization   - **Coherence between the evidence and recommendations:** - Directness of evidence - Consistency of results between studies; in case of inconsistency considered judgment was applied - Conclusions are supported by data - Clinical relevance of conclusions - Conclusions derived from data point to effectiveness/ineffectiveness of the intervention and the recommendation - Justification of (Non)-recommendation in case of weak evidence - Adequate description of the hierarchy of evidence   🡪 no further operationalization   - **Overall assessment of recommendation with regard to risk of bias:** - Adequate description and justification of the strength of evidence - Risks and benefits have been weighted   🡪 no further operationalization | - Item 9. The strengths and limitations of the body of evidence are clearly described.   **Descriptions** of how the body of evidence was evaluated for bias and how it was interpreted by members of the guideline development group   - study design(s) - study methodology limitations (sampling, blinding, allocation concealment, analytical methods) - appropriateness/relevance of primary and secondary outcomes considered - consistency of results across studies - direction of results across studies - magnitude of benefit versus magnitude of harm - applicability to practice context   🡪 no further operationalization | - Item 16: Is the quality of evidence that supports each recommendation stated explicitly? | - Assessment of the critical appraisal of the included studies and its documentation. - No predefined appraisal criteria, only examples given   🡪 no further operationalization   - Assessment of the appropriate consideration of study quality to formulate conclusions. - 🡪 no further operationalization - Assessment of the appropriateness of methods used to combine the findings of studies   🡪 no further operationalization | - Assessment of the provided information regarding: - Method of data extraction - Critical appraisal method - Methods for data synthesis - Presentation of results of the assessment - Assessment of the consideration of: - Medico-legal implications - Economic aspects - Ethical implications - Social implications - Other perspectives (stakeholder, patients, consumers) |
| **Assessment of the consensus process** | | | | |
| - s. AGREE (no assessment beyond this) | - Item 10. The methods for formulating the recommendations are clearly described:   Item content includes the following criteria*:*   - description of the recommendation development process (e.g., steps used in modified Delphi technique, voting procedures that were considered) - outcomes of the recommendation development process (e.g., extent to which consensus was reached using modified Delphi technique, outcome of voting procedures) - description of how the process influenced the recommendations (e.g., results of Delphi technique influence final recommendation, alignment with recommendations and the final vote)   Additional considerations:   - Was a formal process used to arrive at the recommendations? - Were the methods appropriate?   🡪 no further operationalization   - **Domain 6: Editorial independence** - Item 22: The views of the funding body have not influenced the content of the guideline. - Item 23:. Competing interests of guideline development group members have been recorded and addressed. | No assessment of the consensus process | n. a. | n. a. |
| *: operationalization for systematic reviews is not stated due to focus of this paper on CPG  CPG: clinical practice guideline  CI: confidence interval  HTA Health technology assessment  n. a.: not applicable | | | | |

**Data extraction was based on the following sources:**
